# Smoking-mediated up-regulation of GAD67 expression in the human airway epithelium

**DOI:** 10.1186/1465-9921-11-150

**Published:** 2010-10-29

**Authors:** Guoqing Wang, Rui Wang, Barbara Ferris, Jacqueline Salit, Yael Strulovici-Barel, Neil R Hackett, Ronald G Crystal

**Affiliations:** 1 Department of Genetic Medicine, Weill Cornell Medical College, New York, New York, USA

## Abstract

**Background:**

The production of gamma-amino butyric acid (GABA) is dependent on glutamate decarboxylases (GAD65 and GAD67), the enzymes that catalyze the decarboxylation of glutamate to GABA. Based on studies suggesting a role of the airway epithelial GABAergic system in asthma-related mucus overproduction, we hypothesized that cigarette smoking, another disorder associated with increased mucus production, may modulate GABAergic system-related gene expression levels in the airway epithelium.

**Methods:**

We assessed expression of the GABAergic system in human airway epithelium obtained using bronchoscopy to sample the epithelium and microarrays to evaluate gene expression. RT-PCR was used to confirm gene expression of GABAergic system gene in large and small airway epithelium from heathy nonsmokers and healthy smokers. The differences in the GABAergic system gene was further confirmed by TaqMan, immunohistochemistry and Western analysis.

**Results:**

The data demonstrate there is a complete GABAergic system expressed in the large and small human airway epithelium, including glutamate decarboxylase, GABA receptors, transporters and catabolism enzymes. Interestingly, of the entire GABAergic system, smoking modified only the expression of GAD67, with marked up-regulation of GAD67 gene expression in both large (4.1-fold increase, p < 0.01) and small airway epithelium of healthy smokers (6.3-fold increase, p < 0.01). At the protein level, Western analysis confirmed the increased expression of GAD67 in airway epithelium of healthy smokers compared to healthy nonsmokers (p < 0.05). There was a significant positive correlation between GAD67 and MUC5AC gene expression in both large and small airway epithelium (p < 0.01), implying a link between GAD67 and mucin overproduction in association with smoking.

**Conclusions:**

In the context that GAD67 is the rate limiting enzyme in GABA synthesis, the correlation of GAD67 gene expression with MUC5AC expressions suggests that the up-regulation of airway epithelium expression of GAD67 may contribute to the increase in mucus production observed in association with cigarette smoking.

**Trial registration:**

NCT00224198; NCT00224185

## Background

Gamma-aminobutyric acid (GABA) is a multifunctional mediator that functions as a neurotransmitter in the central nervous system and a trophic factor during nervous system development, affecting proliferation, differentiation and cell death [1-3]. GABA is synthesized from glutamate, and catalyzed by GAD65 and GAD67, glutamic acid decarboxylase [1-3]. In the CNS, transporters, receptors and catabolic enzymes work in a coordinated fashion to control the availability of GABA [1-3]. It is now recognized that GABA also functions in a variety of organs outside of the CNS [1,3,4]. In the lung, a series of recent studies suggest that the GABAergic signaling system plays a role in the control of asthma-related airway constriction and mucin secretion [5-9].

In the context that goblet cell hyperplasia and mucin overproduction is also associated with cigarette smoking [10-12], we hypothesized that components of the GABAergic system may also be altered in the airway epithelium of cigarette smokers. To assess this hypothesis, we examined our microarray database of large and small airway gene expression of healthy nonsmokers and healthy smokers to determine if the GABAergic system was expressed. This was verified by PCR analysis. The data demonstrate there is expression of genes for a complete GABAergic system in the airway epithelium. Interestingly, the expression of GAD67 was markedly modified by smoking, with increased expression in healthy smokers compared to healthy nonsmokers at the mRNA and protein levels. In the context that mucus overproduction is commonly associated with cigarette smoking, GAD67 may be a pharmacologic target for the treatment of smoking-related disorders.

## Methods

### Study Population

Healthy nonsmokers and healthy smokers were recruited using local print media. The study population was evaluated at the Department of Genetic Medicine Clinical Research Facility under the auspices of the Weill Cornell NIH Clinical and Translational Science Center with approval by the Weill Cornell Medical College Institutional Review Board. Written informed consent was obtained from each volunteer before enrollment in the study. Individuals were determined to be phenotypically normal on the basis of clinical history and physical examination, routine blood screening tests, urinalysis, chest X-ray, ECG and pulmonary function testing. Current smoking status was confirmed by history, venous carboxyhemoglobin levels and urinalysis for levels of nicotine and its derivative cotinine. All individuals were asked not to smoke for at least 12 hr prior to bronchoscopy.

### Collection of Airway Epithelial Cells

Epithelial cells from the large and small airways were collected using flexible bronchoscopy. After achieving mild sedation and anesthesia of the vocal cords, a flexible bronchoscope (Pentax, EB-1530T3) was advanced to the desired bronchus. Large airway epithelial samples were collected by gentle brushing of the 3^rd ^to 4^th ^order bronchi and small airway samples were collected from 10^th ^to 12^th ^order bronchi using methods previously described [13]. The large and small airway epithelial cells were subsequently collected separately in 5 ml of LHC8 medium (GIBO, Grand Island, NY). An aliquot of this was used for cytology and differential cell count and the remainder was processed immediately for RNA extraction. Total cell counts were obtained using a hemocytometer, whereas differential cell counts were determined on sedimented cells prepared by centrifugation (Cytospin 11, Shandon Instruments, Pittsburgh, PA) and stained with DiffQuik (Baxter Healthcare, Miami, FL).

### RNA Extraction and Microarray Processing

The HG-U133 Plus 2.0 microarray (Affymetrix, Santa Clara, CA), which includes probes for more than 47,000 transcripts genome-wide, was used to evaluate gene expression. Total RNA was extracted using a modified version of the TRIzol method (Invitrogen, Carlsbad, CA), in which RNA is purified directly from the aqueous phase (RNeasy MinElute RNA purification kit, Qiagen, Valencia, CA). RNA samples were stored in RNA Secure (Ambion, Austin, TX) at -80°C. RNA integrity was determined by running an aliquot of each RNA sample on an Agilent Bioanalyzer (Agilent Technologies, Palo Alto, CA). The concentration was determined using a NanoDrop ND-1000 spectrophotometer (NanoDrop Technologies, Wilmington, DE). Double-stranded cDNA was synthesized from 1 to 2 μg total RNA using the GeneChip One-Cycle cDNA Synthesis Kit, followed by cleanup with GeneChip Sample Cleanup Module, *in vitro *transcription (IVT) reaction using the GeneChip IVT Labeling Kit, and cleanup and quantification of the biotin-labeled cDNA yield by spectrophotometry. All kits were from Affymetrix (Santa Clara, CA). All HG-U133 Plus 2.0 microarrays were processed according to Affymetrix protocols, hardware and software, including being processed by the Affymetrix fluidics station 450 and hybridization oven 640, and scanned with an Affymetrix Gene Array Scanner 3000 7G. Overall microarray quality was verified by the following criteria: (1) RNA Integrity Number (RIN) ≥7.0; (2) 3'/5' ratio for GAPDH ≤3; and (3) scaling factor ≤10.0.

### Microarray Data Analysis

Captured images were analyzed using Microarray Suite version 5.0 (MAS 5.0) algorithm (Affymetrix) as previously described [13-15]. The data were normalized using GeneSpring version 7.0 software (Agilent Technologies, Palo Alto, CA) as follows: (1) per array, by dividing raw data by the 50^th ^percentile of all measurements; and (2) per gene, by dividing the raw data by the median expression level for all the genes across all arrays in a dataset.

### RT-PCR

To confirm the expression of the genes in the GABAergic system, total RNA from large airway epithelium and small airway epithelium was prepared as described above. Total RNA from whole human brain (Clontech, Mountain View, CA) was used as a positive control. RNA was reverse transcribed by TaqMan Reverse Transcription Regents (ABI, Foster City, CA). Routine PCR was performed using Platinum PCR Supermix (Invitrogen, Carlsbad, CA) at indicated temperatures and times (Additional file 1, Table S1).

### TaqMan RT-PCR Confirmation of Microarray Expression Levels

To quantify relative mRNA levels of GAD67, TaqMan real-time RT-PCR was performed on a random sample of large and small airway samples of 10 healthy nonsmokers and 12 healthy smokers that had been used for the HG-U133 Plus 2.0 microarray analyses. First, cDNA was synthesized from 2 μg RNA in a 100 μl reaction volume, using the Reverse Transcriptase Reaction Kit (Applied Biosystems), with random hexamers as primers. Dilutions of 1:10 and 1:100 were made from each sample and triplicate wells were run for each dilution. TaqMan PCR reactions were carried out using pre-made kits from Applied Biosystems and 2 μl of cDNA was used in each 25 μl reaction volume. β-actin was used as the endogenous control, and relative expression levels were determined using the ΔΔCt method (Applied Biosystems). The β-actin probe was labeled with VIC and the probe for GAD67 with FAM. The PCR reactions were run in an Applied Biosystems Sequence Detection System 7500.

### Localization of GAD67 Expression in Human Airway Epithelium

To determine the airway epithelial localization of GAD67 expression, bronchial biopsies were obtained by flexible bronchoscopy from the large airway epithelium of 10 healthy nonsmokers and 10 healthy smokers [13]. Immunohistochemistry was carried out on these paraffin-embedded endobronchial biopsies. Sections were deparaffinized and rehydrated through a series of xylenes and alcohol. To enhance staining, an antigen retrieval step was carried out by boiling the sections at 100°C, 20 min in citrate buffer solution (Labvision, Fremont, CA), followed by cooling at 23°C, 20 min. Endogenous peroxidase activity was quenched using 0.3% H_2_O_2_, and blocking was performed with normal goat serum to reduce background staining. Samples were incubated with the mouse monoclonal anti-GAD67 antibody (1 μg/μl at 1/25 dilution, Millipore, Billerica, MA), 16 hr, 4°C. Cytospin slides of 293 cells transfected with pcDNA3.1-GAD67, and pcDNA3.1 plasmids were used as controls. Vectastain Elite ABC kit (Vector Laboratories, Burlingame, CA) and 3-amino-9-ethylcarbazole (AEC) substrate kit (Dako, Carpinteria, CA) were used to detect antibody binding, and the sections were counterstained with hematoxylin (Sigma-Aldrich, St. Louis, MO), and mounted using GVA mounting medium (Zymed, San Francisco, CA). Brightfield microscopy was performed using a Nikon Microphot microscope and images were captured with an Olympus DP70 CCD camera.

### Western Analysis

Western analysis was used to quantitatively assess GAD67 protein expression in small airway epithelium from healthy nonsmokers and healthy smokers. Brushed small airway epithelial cells were obtained as described above. Initially, the cells were centrifuged at 600 g, 5 min, 4°C. The whole cells were lysed with red cell lysis buffer (Sigma-Aldrich), followed by whole cell lysis buffer (ACK lysing buffer, Invitrogen), and then protease inhibitor (Cell Lytic Mammalian Tissue Lysis/Extraction reagent, Sigma-Aldrich) was added to the sample. The sample was centrifuged at 10,000 g and the protein-containing supernatant was collected. The protein concentrations were assessed using a bicinchoninic acid (BCA) protein concentration kit (Pierce, Rockford, IL). Equal concentration of protein (20 μg), mixed with SDS Sample Loading Buffer (Bio-Rad, Hercules, CA) and reducing agent, was loaded on Tris-glycine gels (Bio-Rad). Protein electrophoresis was carried out at 100 V, 2 hr, 23°C. Sample proteins were transferred (25 V, 1 hr, 4°C) to a 0.45 μm PVDF membrane (Invitrogen) using Tris-glycine transfer buffer (Bio-Rad). After transfer the membranes were blocked with 5% milk in PBS for 1 hr, 23°C. The membranes were incubated with primary mouse monoclonal anti-GAD67 antibody (Millipore, Billerica, MA) at 1:2000 dilution, 2 hr, 4°C. Protein extracted from pcDNA 3.1-GAD67 transfected 293 cells was used as a positive control. Detection was performed using horseradish peroxidase-conjugated anti-mouse antibody (1:10,000 dilution, Santa Cruz Biotechnology, Santa Cruz, CA) and the Enhanced Chemiluminescent reagent (ECL) system (GE, Healthcare, Pittsburgh, PA) using Hyperfilm ECL (GE Healthcare). The membrane was subsequently stripped and reincubated with horseradish peroxidase-conjugated anti-β-actin antibody (Santa Cruz Biotechnology) as a control for equal protein concentration. To assess the Western analyses quantitatively, the film was digitally imaged, maintaining exposure within the linear range of detection. The contrast was inverted, the pixel intensity of each band determined, and the background pixel intensity for a negative area of the film of identical size subtracted using MetaMorph image analysis software (Universal Imaging, Downingtown, PA).

### MUC5AC Staining

For MUC5AC staining in large airway and small airway epithelium, brush cells cytospin slides were stained with mouse anti-human MUC5AC antibody (Vector, Burlingame, CA) and detected by Cy3 labeled goat anti-mouse antibody (Jackson, West Grove, PA). Nuclei were counterstained with DAPI (Invitrogen, Carlsbad, CA). Based on the microarray data, we defined "high GAD67" or "high MUC5AC" gene expression as ≥median + 1 standard deviation and low GAD67 or low MUC5AC gene expression as ≤median - 1 standard deviation. Based on this criteria, 3 healthy smokers with high GAD67 and high MUC5AC gene expression and 3 healthy smokers with low GAD67 and low MUC5AC gene expression were assessed for MUC5AC protein expression by immunofluorescence staining.

### Statistical Analysis

HG-U133 Plus 2.0 microarrays were analyzed using GeneSpring software. Average expression values for GAD67 in large and small airway samples (HG-U133 Plus 2.0) were calculated from normalized expression levels for nonsmokers and healthy smokers. Statistical comparisons for microarray data were calculated using GeneSpring software and associated two-tailed Students t-test. Benjamini-Hochberg correction was applied to limit the false discovery rate. Statistical comparisons for categorical data were achieved using Chi-squared test. Correlations were performed using Pearson correlation. All other statistical comparisons were calculated using a two-tailed (Welsh) t-test.

### Web Deposition of Data

All data has been deposited in the Gene Expression Omnibus (GEO) site (http://www.ncbi.nlm.nih.gov/geo), curated by the National Center for Bioinformatics. Accession number for the data is GSE17905.

## Results

### Study Population

Large airway samples from 21 healthy nonsmokers and 31 healthy smokers and small airway samples from a total of 105 individuals, including 47 healthy nonsmokers and 58 healthy smokers, were analyzed with Affymetrix HG-U133 Plus 2.0 microarray (Table 1). All healthy individuals had no significant prior medical history, no history suggestive of asthma and a normal general physical examination. There were no differences between groups with regard to ancestral background (p > 0.05). For the large airways and small airway, there were no gender difference (p > 0.5), and no age difference (p > 0.1), between the nonsmoker and smoker groups. All individuals were HIV negative, with blood and urine parameters within normal ranges (p > 0.05 for all comparisons). Urine nicotine and cotinine, and venous blood carboxyhemoglobin levels of smokers confirmed current smoking status of these individuals. Pulmonary function testing, with and without bronchodilators, revealed normal lung function in healthy nonsmokers and all healthy smokers (Table 1).

**Table 1:** Study Population of Airway Epithelial Samples ^1^

	Large airways		Small airways	
	
Parameter	Healthy nonsmokers	Healthy smokers	Healthy nonsmokers	Healthy smokers
n	21	31	47	58
Sex (male/female)	15/6	21/10	33/14	38/20
Age (yr)	41 ± 8	44 ± 7	42 ± 11	43 ± 7
Race (B/W/O)^2^	10/7/4	20/7/4	23/18/6	35/14/9
Smoking history (pack-yr)	0	28 ±18	0	28 ± 17
Urine nicotine (ng/ml)	Negative	746 ± 904	Negative	1298 ±1692
Urine cotinine (ng/ml)	Negative	973 ± 690	Negative	1246 ± 974
Venous CO-Hb^3^	0.64 ± 0.93	2.0 ±1.9	0.4 ± 0.8	1.8 ± 1.9
Pulmonary function^4^				
FVC	106 ± 13	110 ± 11	107 ± 14	109 ± 13
FEV1	107 ± 17	110 ± 12	106 ± 15	107 ± 14
FEV1/FVC	82 ± 5	81 ± 5	82 ± 6	80 ± 5
TLC	100 ± 14	103 ± 11	101 ±13	100 ±12
DLCO	101 ± 16	95 ± 11	99 ± 15	94 ± 11
Epithelial cells				
Total number × 10^6^	7.0 ± 3	7.0 ± 3.3	6.3 ± 2.9	7.2 ± 3.0
% epithelial	99.7 ± 0.6	99.8 ± 0.5	99.3 ± 1.1	99.1 ± 1.3
% inflammatory	0.3 ± 0.6	0.2 ± 0.5	0.7 ± 1.1	0.8 ± 1.3
Differential cell count (%)				
Ciliated	53.6 ± 6.6	47.8 ± 13.7	74.3 ± 7.4	65.7 ± 12.5
Secretory	10 ± 4.4	10 ± 4.1	6.6 ± 3.5	9.1 ± 4.5
Basal	22.4 ± 3.4	25.9 ± 9.9	11.1 ± 5.3	12.7 ± 6.7
Undifferentiated	14.1 ± 5.2	16.5 ± 8.9	7.3 ± 3.2	11.8 ± 6.7

^1^Data are presented as mean ∀ standard deviation.

^2^B = Black, W = White, O = Other.

^3^Venous carboxyhemoglobin, a secondary marker of current smoking; nonsmokers, normal value <1.5%.

^4^Pulmonary function testing parameters are given as % of predicted value with the exception of FEV1/FVC, which is reported as % observed; FVC - forced vital capacity, FEV1 - forced expiratory volume in 1 sec, TLC - total lung capacity, DLCO - diffusing capacity.

### Sampling of Airway Epithelium

Airway epithelial cells were obtained by fiberoptic bronchoscopy and brushing of the large (3^rd ^to 4^th ^order) and small (10^th ^to 12^th ^order) airways. The number of cells recovered ranged from 6.3 to 7.2 × 10^6 ^(Table 1). The percent epithelial cells recovered was, on average, 99% in all groups. The various categories of airway epithelial cells were, as expected, from the large and small airways [13,15].

### Expression of GABAergic System-related Genes in the Airway Epithelium

**Figure 1:**
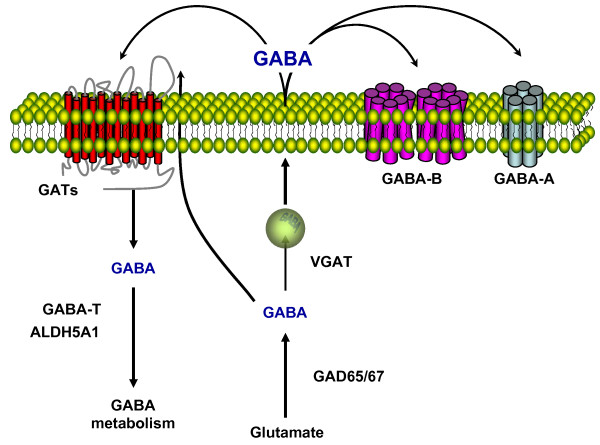
**Schematic illustration of GABAergic system**. GABA is synthesized from glutamate by the glutamic acid decarboxylases GAD67 and GAD65. GABA is released by either a vesicle-mediated process, a vesicular neurotransmitter transporter (VGAT) or a nonvesicular process by reverse transport. GABA exerts its physiological effects through GABA-A and GABA-B receptors. The GABAergic signal is terminated by rapid uptake of GABA by specific high affinity GABA transporters (GATs). There are 4 distinct genes encoding GABA membrane transporters, GAT-1, GAT-2, GAT-3 and BGT-1. GABA is metabolized by GABA transaminase (GABA-T) and succinic semialdehyde dehydrogenase (ALDH5A1).

Based on the function in GABAergic system, we categorized GABAergic system-related genes into 4 groups: synthesis, receptor, transport, metabolism (Figure 1, Table 2). Synthesis-related genes include GAD65 and GAD67; receptor-related genes include 19 GABA-A receptor subunits (alpha 1-6, beta 1-3, epsilon, gamma 1-3, pi, theta, delta, rho1-3) and 2 GABA-B receptor subunits (GABBR1, GABBR2). Transport-related genes include GABA vesicular transporter (VGAT), GABA transporter 1 (GAT-1), GAT-2, GAT-3, Na(+)/Cl(-) betaine/GABA transporter (BGT-1). Metabolism-related genes include GABA transferase (GABA-T) and aldehyde dehydrogenase 5 family, member A1 (ALDH5A1).

**Table 2:** Expression of GABAergic System Genes in Large Airway and Small Airway Epithelium of Healthy Smokers Compared to Healthy Nonsmokers^1^

			**Large airway(smoker/nonsmoker)**^**2**^			**Small airway (smoker/nonsmoker)**^**2**^		
			
Probe set ID	Gene symbol	Gene title	Fold-change	**p value**^**3**^	**P call (%)**^**4**^	Fold-change	**p value**^**3**^	**P call (%)**^**4**^
**Synthesis**								
206780_at	GAD65	glutamate decarboxylase 2	1.08	0.86	0.0	1.05	0.89	0.0
205278_at	GAD67	glutamate decarboxylase 1	4.09	2.07 × 10^-5^	80.8	6.27	2.33 × 10^-11^	59.0
**Receptor**								
244118_at	GABRA1	gamma-aminobutyric acid (GABA) A receptor, alpha 1	-1.27	0.60	1.9	1.12	0.84	1.9
207014_at	GABRA2	gamma-aminobutyric acid (GABA) A receptor, alpha 2	-1.26	0.60	0.00	1.12	0.83	1.0
207210_at	GABRA3	gamma-aminobutyric acid (GABA) A receptor, alpha 3	1.80	0.16	0.00	1.17	0.69	1.0
208463_at	GABRA4	gamma-aminobutyric acid (GABA) A receptor, alpha 4	1.21	0.63	7.7	1.05	0.89	6.7
215531_s_at	GABRA5	gamma-aminobutyric acid (GABA) A receptor, alpha 5	-1.46	0.46	1.9	1.01	0.95	1.0
207182_at	GABRA6	gamma-aminobutyric acid (GABA) A receptor, alpha 6	1.07	0.91	0.0	1.49	0.20	0.0
207010_at	GABRB1	gamma-aminobutyric acid (GABA) A receptor, beta 1	1.38	0.57	9.6	-1.14	0.81	6.7
242344_at	GABRB2	gamma-aminobutyric acid (GABA) A receptor, beta 2	1.34	0.33	61.5	1.15	0.70	41.9
229724_at	GABRB3	gamma-aminobutyric acid (GABA) A receptor, beta 3	-1.01	0.98	96.2	1.10	0.81	97.1
241805_at	GABRG1	gamma-aminobutyric acid (GABA) A receptor, gamma 1	1.09	0.86	15.4	-1.11	0.82	33.3
1568612_at	GABRG2	gamma-aminobutyric acid (GABA) A receptor, gamma 2	1.02	0.96	0.0	1.34	0.49	0.0
216895_at	GABRG3	gamma-aminobutyric acid (GABA) A receptor, gamma 3	-1.10	0.86	9.6	-1.74	0.14	14.3
204537_s_at	GABRE	gamma-aminobutyric acid (GABA) A receptor, epsilon	1.13	0.56	98.1	-1.29	0.12	72.4
220886_at	GABRQ	gamma-aminobutyric acid (GABA) receptor, theta	1.34	0.56	1.9	-1.03	0.91	1.0
230255_at	GABRD	gamma-aminobutyric acid (GABA) A receptor, delta	1.15	0.51	0.0	-1.02	0.91	0.0
5044_at	GABRP	gamma-aminobutyric acid (GABA) A receptor, pi	1.08	0.70	100.0	-1.07	0.81	100.0
206525_ at	GABRR1	gamma-aminobutyric acid (GABA) receptor, rho 1	1.81	0.29	23.1	-1.27	0.53	15.2
208217_at	GABRR2	gamma-aminobutyric acid (GABA) receptor, rho 2	-1.12	0.73	11.5	1.24	0.20	21.9
234410_at	GABRR3	gamma-aminobutyric acid (GABA) receptor, rho 3	1.09	0.86	0.0	1.41	0.26	1.0
205890_s_at	GABBR1	gamma-aminobutyric acid (GABA) B receptor, 1	-1.45	0.31	94.2	-1.75	1.79 × 10^-4^	98.1
209990_s_at	GABBR2	gamma-aminobutyric acid (GABA) B receptor, 2	-1.09	0.86	15.4	-1.33	0.42	7.6
**Transport**								
205152_at	GAT-1	solute carrier family 6 (neurotransmitter transporter, GABA), member 1	-1.37	0.46	26.9	-1.70	0.14	44.7
237058_x_at	GAT-2	solute carrier family 6 (neurotransmitter transporter, GABA), member 13	-1.35	0.30	100.0	-1.13	0.60	99.1
207048_at	GAT-3	solute carrier family 6 (neurotransmitter transporter, GABA), member 11	-1.09	0.86	1.9	1.14	0.69	1.0
206058_at	BGT-1	solute carrier family 6 (neurotransmitter transporter, betaine/GABA), member 12	-1.18	0.70	17.3	-1.04	0.90	8.6
240532_at	VGAT	solute carrier family 32 (GABA vesicular transporter), member 1	1.03	0.95	0.0	-1.15	0.69	0.0
**Metabolism**								
209460_at	GABA-T	4-aminobutyrate aminotransferase	-1.45	7.25 × 10^-2^	100.0	-1.45	5.41 × 10^-3^	100.0
203608_at	ALDH5A1	aldehyde dehydrogenase 5 family, member A1	-1.18	0.31	100.0	-1.15	0.12	100.0

^1^Data was obtained using the Affymetrix HG-U133 Plus 2.0 microarray chip.

^2^Fold-change represents the ratio of average expression value in healthy smokers to average expression value in healthy nonsmokers. Positive fold-changes represent genes up-regulated by smoking; negative fold-changes represent genes down-regulated by smoking.

^3^p value obtained using Benjamini-Hochberg correction to limit the false positive rate.

^4^P call represents the % of healthy nonsmoker and healthy smoker samples in which the Affymetrix detection call for that probe set was "P" or "Present," i.e., the gene was expressed in that sample.

**Figure 2:**
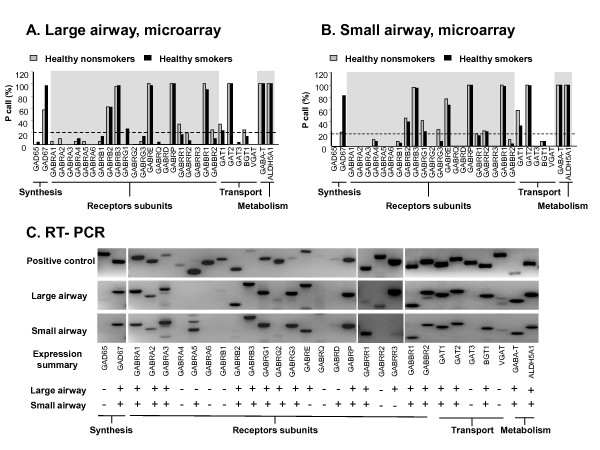
**GABAergic system gene expression in large and small airway epithelium**. **A**. Microarray present call analysis of GABAergic system genes in large airway epithelium. **B**. Microarray present call analysis of GABAergic system genes in small airway epithelium. For **A **and **B**, the dashed line represents P call of 20%. **C**. RT-PCR assessment of GABAergic system gene expression in large and small airway epithelium. Human brain RNA was used as a positive control. Shown are representative RT-PCR results of 1 large airway epithelium sample and 1 small airway epithelium sample.

Of the 30 GABAergic system-related genes surveyed using the Affymetrix HG-U133 Plus 2.0 array and the criteria of Affymetrix Detection Call of Present (P call) in ≥20%, there were 13 GABAergic system genes expressed in the large airway epithelium of healthy nonsmokers and 11 in the large airway epithelium of healthy smokers (Figure 2A, B). The 13 GABAergic genes expressed in the large airway epithelium of nonsmokers included synthesis-related genes GAD67; receptors GABRB2, GABRB3, GABRE, GABRP, GABRR2, GABBR1, GABBR2; transport-related genes GAT-1,GAT-2, BGT-1 and metabolism-related genes GABA-T, ALDH5A1. The 11 GABAergic gene expressed in the large airway epithelium of smokers included synthesis-related genes GAD67; receptors GABRB2, GABRB3, GABRE, GABRG1, GABRP, GABBR1; transport-related genes GAT-1,GAT-2 and metabolism-related genes GABA-T, ALDH5A1. In the small airway epithelium there were 13 GABAergic genes expressed in healthy nonsmokers and 12 GABAergic genes in healthy smokers, respectively (Figure 2A, B). The 13 GABAergic genes expressed in the small airway epithelium of nonsmokers included synthesis-related genes GAD67; receptors GABRB2, GABRB3, GABRG1, GABRG3, GABRE, GABRP, GABRR2, GABBR1; transport-related genes GAT-1,GAT-2 and metabolism-related genes GABA-T, ALDH5A1. The 12 GABAergic gene expressed in the small airway epithelium of smokers included synthesis-related genes GAD67; receptors GABRB2, GABRB3, GABRE, GABRG1, GABRP, GABRR2, GABBR1; transport-related genes GAT-1,GAT-2 and metabolism-related genes GABA-T, ALDH5A1.

Independent of smoking status, the only GABA synthesis enzymes expressed in the large airway epithelium and small airway epithelium was GAD67. In regard to transporters, there was no GAT-3 and VGAT expression in human large and small airway epithelium. For the GABA metabolism-related genes, both GABAT and ALDH5A1 were expressed in the large and small airway epithelium. In summary, each functional group of the GABA system has genes expressed in airway epithelium, forming a complete GABAergic system. RT-PCR confirmed that a complete GABAergic system was expressed in the airway epithelium (Figure 2C).

### Up-regulation of GAD67 in Large and Small Airway Epithelium of Healthy Smokers

**Figure 3:**
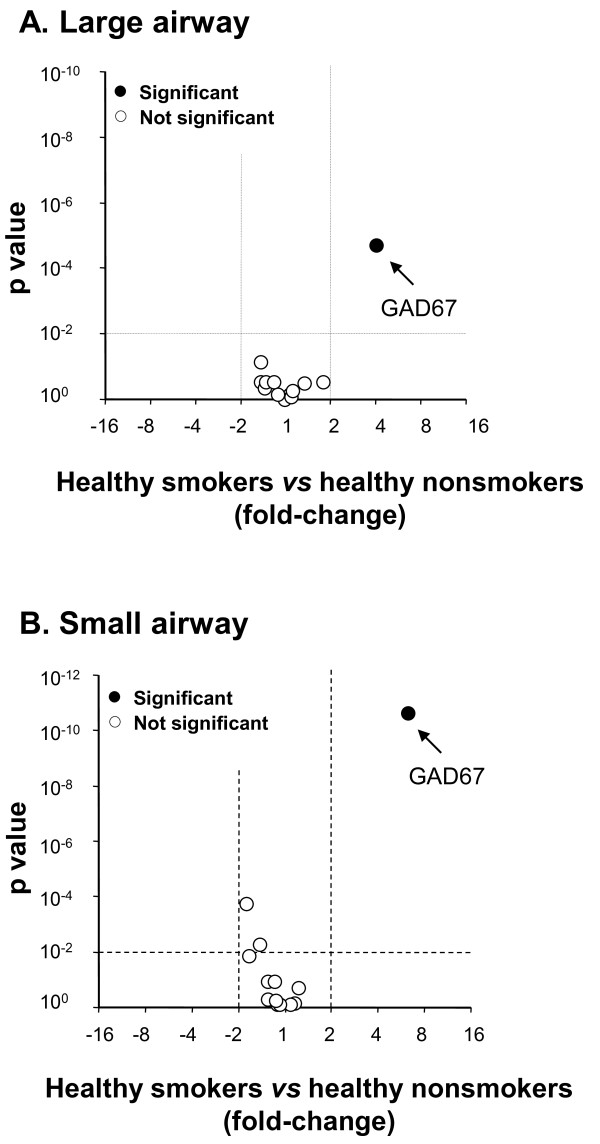
**Microarray assessment of smoking-induced change in GABAergic system gene expression in large and small airway epithelium**. **A**. Volcano plot of GABAergic system gene-related probe sets in large airway epithelium. **B**. Volcano plot of GABAergic system gene-related probe sets in small airway epithelium. For both panels, the x-axis corresponds to the fold-change and the y-axis corresponds to p value. Black dots represent significant differentially expressed probe sets; open dots represent probe sets with no significant difference between healthy smokers and healthy nonsmokers. The changes in gene expression were considered significant based on the criteria of fold-change >2, p < 0.01, with Benjamini-Hochberg correction

**Figure 4:**
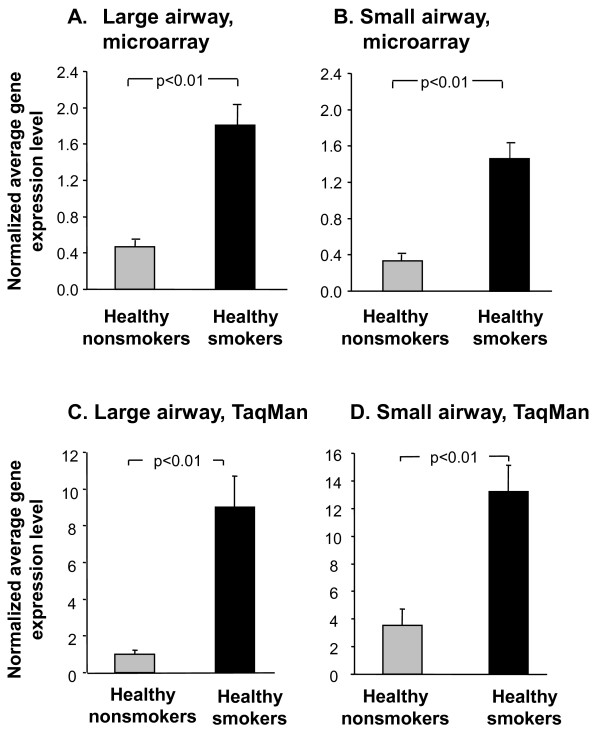
**GAD67 gene expression levels in large and small airway epithelium of healthy smokers compared to healthy nonsmokers**. **A**. Average normalized gene expression levels of GAD67, assessed using HG-U133 Plus 2.0 microarray in large airway epithelium of 21 healthy nonsmokers and 31 healthy smokers. The ordinate shows the average normalized gene expression levels for GAD67. **B**. Average normalized gene expression levels of GAD67, assessed using HG-U133 Plus 2.0 microarray in small airway epithelium of 47 healthy nonsmokers and 58 healthy smokers. **C**. TaqMan confirmation of changes in GAD67 gene expression levels in large airways of 10 healthy nonsmokers and 12 healthy smokers. **D**. TaqMan confirmation of changes in GAD67 gene expression levels in small airways of 10 healthy nonsmokers and 12 healthy smokers. The ordinate shows average gene expression levels and error bars represent standard error.

Of all of the GABAergic system genes expressed in the large and small airways, only GAD67 was significantly changed >2-fold in healthy smokers compared to healthy nonsmokers (Figure 3A, B). As assessed using the microarrays, GAD67 was significantly up-regulated in healthy smokers compared to healthy nonsmokers in the large airway epithelium (4.1-fold increase, p < 0.01; Figure 4A), and healthy smokers compared to healthy nonsmokers in the small airway epithelium (6.3-fold increase, p < 0.01; Figure 4B). To confirm the results obtained from the microarray screen, TaqMan RT-PCR was carried out on RNA samples from the large and small airways epithelium of 10 healthy nonsmokers and 12 healthy smokers, respectively. The TaqMan data confirmed that GAD67 was significantly up-regulated in the large airways of healthy smokers (8.8-fold increase, p < 0.01) compared to healthy nonsmokers (Figure 4C), and in the small airways of healthy smokers (3.8-fold increase, p < 0.01) compared to healthy nonsmokers (Figure 4D). Interestingly, when human airway epithelial cell line 16HBE was treated with cigarette smoking extract *in vitro*, GAD67 gene expression was also up-regulated (not shown).

### Immunohistochemical Assessment of GAD67 Expression

**Figure 5:**
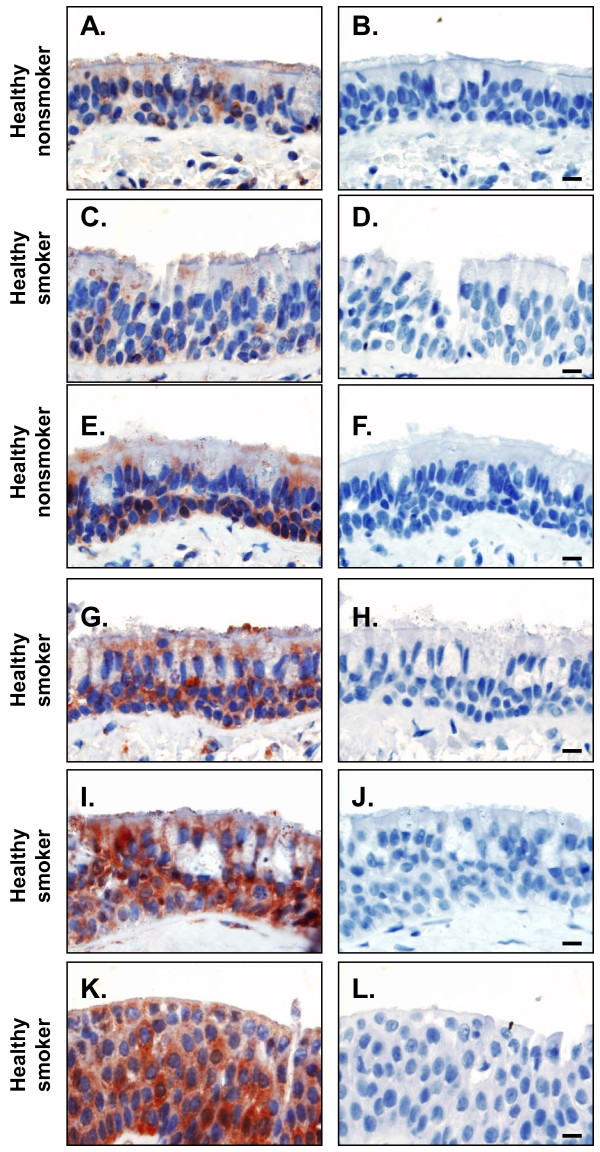
**Immunohistochemistry assessment of GAD67 expression in large airway epithelium in healthy nonsmokers and healthy smokers, representing the broad range of up-regulation of the GAD67 gene**. Panel**s A, C, E, G, I, K**, stained with anti-GAD67 antibody. Panels **B, D, F, H, J, L**, stained with mouse IgG control. **A**. Healthy nonsmoker, anti-GAD67; **B**. Healthy nonsmoker, IgG; **C**. Healthy smoker, anti-GAD67; **D**. Healthy smoker, IgG; **E**. Healthy nonsmoker, anti-GAD67; **F**. Healthy nonsmoker, IgG; **G**. Healthy smoker, anti-GAD67; **H**. Healthy smoker, IgG; **I**. Healthy smoker, anti-GAD67; **J**. Healthy smoker, IgG; **K**. Healthy smoker, anti-GAD67; and **L**. Healthy smoker, IgG l. Bar = 10 μm.

The GAD67 expression was assessed at the protein level with immunohistochemistry evaluation of endobronchial biopsy specimens from the large airways of healthy nonsmokers and healthy smokers. The specificity of the anti-GAD67 monoclonal antibody was assessed in 293 cells transfected with the human GAD67 cDNA. Only GAD67 transfected cells were GAD67 positive, while control plasmids transfected cells were GAD67 negative (not shown). In the airway epithelium, positive staining for GAD67 was mainly observed in the basal cell population, but also in ciliated cells (Figure 5). Consistent with our microarray data, there was a variability of GAD67 staining in smokers, with expression ranging from similar to that of healthy nonsmokers (compared panel C to A) to intense GAD67 expression (panels G, I). However, there was much more GAD67 staining overall in the airways epithelium of healthy smokers compared to healthy nonsmokers. Interestingly, squamous metaplasia also showed strong GAD67 staining (panel K).

### Western Analysis of GAD67 Protein Expression

**Figure 6:**
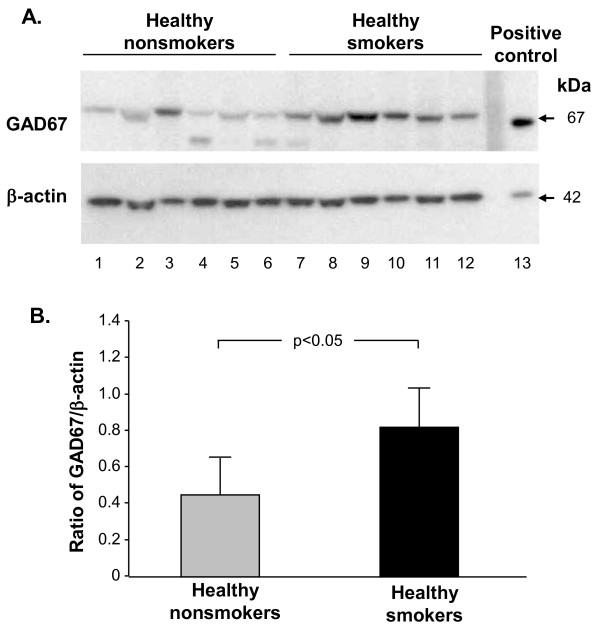
**Western analysis of GAD67 protein expression in small airway epithelium of healthy nonsmokers and healthy smokers**. **A**. Upper panel - GAD67 protein expression in nonsmokers (lanes 1-6), smokers (lanes 7-12) and positive control (lane 13). Lower panel - same gel probed with anti β-actin antibody; 20 μg protein loaded per well. **B**. Ratio of GAD67 to β-actin. The ratio of GAD67 to β-actin is represented on the ordinate for smoker and nonsmoker bands. Error bars represent the standard error. Note in panel **A**, the variability in relative up-regulation of GAD67 in the smokers, similar to that observed at the mRNA level and with immunohistochemistry.

Western analysis carried out on small airway epithelial samples from healthy nonsmokers and healthy smokers was used to quantitatively assess GAD67 protein expression. This analysis confirmed the increased GAD67 protein expression in healthy smokers compared to healthy nonsmokers (p < 0.05, Figure 6).

### Association Between GAD67 and MUC5AC Gene Expression in Smokers

**Figure 7:**
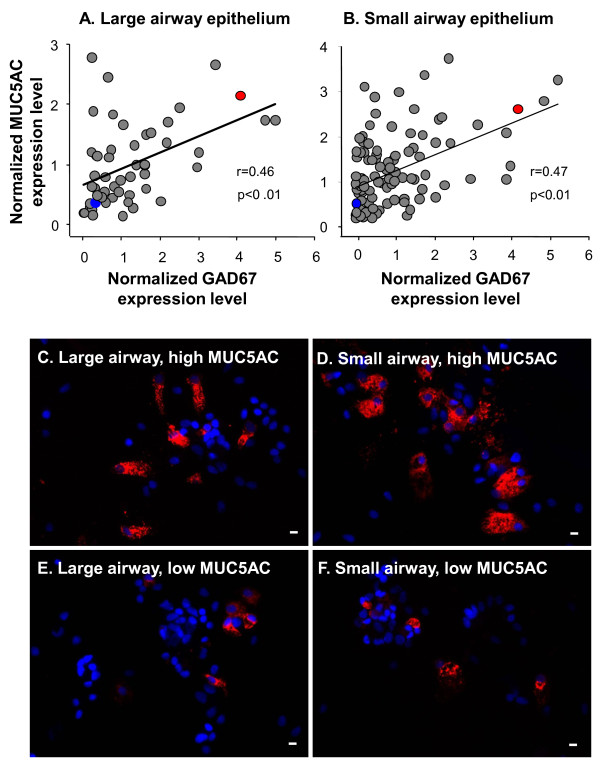
**Association of GAD67 gene expression and MUC5AC expression**. **A, B**. Correlation between GAD67 and MUC5AC gene expression in the large and small airway epithelium (Pearson's correlation). **A**. Average normalized gene expression levels of GAD67 *vs *MUC5AC gene expression in the large airway epithelium. **B**. Average normalized gene expression levels of GAD67 *vs *MUC5AC gene expression in the small airway epithelium. **C-F**. Representative MUC5AC staining on large and small airway epithelial cells from healthy smokers with high GAD67 and high MUC5AC gene expression or with low GAD67 and low MUC5AC gene expression at mRNA level. "High" or "low" gene expression is defined in Methods and based on microarray data. **C**. MUC5AC staining on large airway brushed cells of healthy smokers with high GAD67 and high MUC5AC gene expression (marked as red solid circles in panel A). **D**. MUC5AC staining on small airway brushed cells of healthy smokers with high GAD67 and high MUC5AC gene expression (marked as red solid circles in panel B). **E**. MUC5AC staining on large airway brushed cells of healthy smokers with low GAD67 and low MUC5AC gene expression (marked as blue solid circles in panel A). **F**. MUC5AC staining on small airway brushed cells of healthy smokers with low GAD67 and low MUC5AC gene expression (marked as blue solid circles in panel B). In all panels, IgG controls showed no MUC5AC staining (not shown). Bar = 10 μm.

It has been suggested that GABA can stimulate mucin production in cultured airway epithelial cells [7]. To investigate the relationship between GAD67 and MUC5AC gene expression (the dominant smoking-responsive mucin gene in the human airway epithelium [11,12,16]), the normalized expression of GAD67 was compared to MUC5AC expression. By this method, known mucus biosynthesis-associated genes [e.g., SPDEF (SAM pointed domain containing ets transcription factor)] were found to be highly correlated with MUC5AC gene expression. Significant positive correlations were observed for GAD67 with MUC5AC gene expression in both large (r = 0.46, p < 0.01, Figure 7A) and small airway epithelium (r = 0.47, p < 0.01, Figure 7B). To further assess this association, MUC5AC protein expression was examined in airway brushed cells from healthy smokers with high GAD67 and high MUC5AC gene expression or with low GAD67 and low MUC5AC expression based on microarray data. Immunofluorescence microscopy demonstrated stronger and more extensive distribution of MUC5AC staining in subjects with high GAD67 and high MUC5AC gene expression (Figure 7C, large airway; Figure 7D, small airway) compared to subjects with low GAD67 and low MUC5AC gene expression (Figure 7E, large airway; Figure 7F, small airway). Consistent with this observation, Western analysis showed increased GAD67 expression in small airway epithelium of healthy smokers and COPD smokers compared to nonsmokers (Additional file 1, Figure S1A, B), with some correlation of MUC5AC and GAD67 protein expression (panel C).

## Discussion

Cigarette smoking is associated with mucus hypersecretion by the airway epithelium [10-12]. While the control of mucus secretion is complex, a role of the GABAergic system has been suggested to mediate, in part, the hypersecretion of mucus associated with asthma [6-9,17]. In the context that cigarette smoking is also associated with mucus hypersecretion, in the present study we asked the question: Does smoking alter the gene expression pattern of GABAergic system genes in the respiratory epithelium? Assessment of our database of airway epithelial gene expression generated by microarrays showed that, while many of the GABAergic system genes are expressed in the human large and small airway epithelium, cigarette smoking is associated with changes in gene expression only of GAD67, a gene controlling the synthesis of GABA [2]. A striking increase in gene expression levels of GAD67 was observed in the large and small airway epithelium of healthy smokers compared to healthy nonsmokers, a finding confirmed at the mRNA level by TaqMan PCR; and at the protein level qualitatively by immunohistochemistry, and quantitatively by Western analysis. There was a positive correlation between GAD67 gene expression and MUC5AC at the mRNA level in both small and large airway epithelium, as well as by MUC5AC staining, suggesting a link between mucus overproduction and GAD67 overexpression in association with smoking.

### GABAergic System

GABA is the major inhibitory neurotransmitter in the mammalian central nervous system [2,3]. In the mammalian brain, GABA is synthesized primarily from glutamate in a reaction that is catalyzed by 2 glutamic acid decarboxylase enzymes, GAD65 and GAD67, coded by different genes [1-3]. GABA is then loaded into synaptic vesicles by a vesicular neurotransmitter transporter (VGAT) and liberated from nerve terminals by calcium-dependent exocytosis. Nonvesicular forms of GABA secretion (e.g., by reverse transporter action) have also been described and are likely important during development [18]. After being released from presynaptic nerve terminals, GABA exerts its physiological effects through ionotropic GABA-A receptors and metabotropic GABA-B receptors [19]. The GABAergic neurotransmission is terminated by rapid uptake of the neurotransmitter from the synaptic cleft into neurons and glial cells by specific high-affinity GABA transporters [20]. There are 4 distinct genes encoding membrane GABA transporters, GAT-1, GAT-2, GAT-3, and BGT-1 [20]. Subsequently, GABA is metabolized by a transamination reaction that is catalyzed by GABA transaminase (GABA-T). Succinic semialdehyde dehydrogenase (ALDH5A1), which helps entry of the GABA carbon skeleton into the tricarboxylic acid cycle, is the final enzyme of GABA catabolism [1]. GABAergic system genes are present not only in the brain, but also in other organs, including liver, kidney, pancreas, testis, oviduct, adrenal, and lung [3,4].

### GABAergic System in the Lung

In the lung, immunohistochemistry studies of the guinea pig trachea has identified GABA in airway epithelium, chondrocytes and connective tissue near smooth muscle [21]. GAD65/67 mRNA has been detected in human and mouse airway epithelium at the mRNA level by RT-PCR and at the protein level by Western analysis and immunohistochemistry [7,22]. GABA and GAD 65/67 are also expressed in mouse pulmonary neuroendocrine cells [23]. Of the 19 GABA-A receptor subunits identified in the mammalian genome, subunits alpha1, pi and delta have been detected in human airway epithelium by Western analysis, subunits beta 2/beta 3 in mouse airway epithelium by immunohistochemistry, and alpha 2, gamma 3, beta 1 and pi in rat airway epithelium by immunohistochemistry [24]. Some GABA-A receptor subunits have also been identified in alveolar epithelial cells [25]. There are different expression patterns of some of the GABA-A receptor subunits during rat lung development [24]. Of the GABA-B receptors, both GABBR1 and GABBR2 subunits mRNA have been detected in human airway epithelium and both subunits have been identified by Western analysis and immunohistochemistry in guinea pig trachea [22]. Using specific agonists, GABA-B receptors coupling to G proteins in general and its specific coupling to the G protein was shown in a human airway epithelial cell line [22]. To our knowledge, there has been no prior assessment of expression of GABA transporters or of GABA catabolism enzymes in the human airway epithelium.

In the present study, we categorized the expression of GABAergic system genes into 4 groups based on their GABA-related function: synthesis, receptor, transport and metabolism. The analysis demonstrated a complete GABAergic system exists in the human large and small airway epithelium, although there are differences compared to the central nervous system. Interestingly, in the human airway epithelium there is no VGAT expression, suggesting GABA is released from airway epithelial cells in a vesicle independent fashion [18]. Consistent with our data, high pressure liquid chromatography demonstrated that GABA could be produced in the guinea pig trachea epithelium [26], and a functional GABA transporter has been demonstrated in cultured human airway epithelial cells [27].

### Modification of GAD67 Expression by Smoking

Recent studies suggest the GABAergic system may have a role in oxidative stress protection in neuron-related cells and airway mucus production [7,28,29]. Our data demonstrate that, while many of the GABAergic system genes are expressed in the human large and small airway epithelium, only GAD67 is modified by cigarette smoking, with a marked increase in gene expression levels of GAD67 in the large and small airway epithelium of healthy smokers compared with healthy nonsmokers, and with a positive correlation between GAD67 and MUC5AC, the major airway mucus-related gene [16]. Considering the important role of ion channels in airway surface water balance [30,31], further studies could be directed to explore the effect of GABAergic system changes affected by smoking on the liquid microenvironment of airway epithelium.

The mechanisms responsible for GAD67 gene expression up-regulation by cigarette smoking remain to be elucidated. It is known that nicotine, by activating nicotinic acetylcholine receptors located on cortical or hippocampal GABAergic interneurons, can up-regulate GAD67 expression via an epigenetic mechanism [32]. Inhibitors of DNA methyltransferases and histone deactylases induce GAD67 expression [33,34]. In contrast to this observation, nicotine suppresses protein levels of GAD isozymes (mainly GAD65) and GABA in pancreatic ductal adenocarcinoma tissue [35]. Interestingly, nuclear factor-kappa B activation through oxidative stress can up-regulate GAD67 expression [36], and the early growth response factor 1-related pathway also mediates GAD67 up-regulation [37]. Glucocorticoid hormones can modulate GAD expression by transcriptional activation of the GAD67 promoter [38]. Finally, GAD can also be regulated at the post-translational level by protein phosphorylation, palmitoylation and cleavage [39]. Together, these findings suggest that cigarette smoking may have a complicated effect on GAD activity.

### GABAergic System and Mucus Overproduction

A variety of observations link the GABAergic system to mucus overproduction. Studies by Xiang et al [7] demonstrated that GABA promotes the proliferation of airway epithelial cells, an effect that was suppressed by a GABA-A receptor antagonist, whereas activation of GABA-A receptors depolarized airway epithelial cells. After exposure to GABA for 6 days, cultured human airway epithelium demonstrated more mucus staining. Moreover, ovalbumin-induced airway goblet cell hyperplasia and mucus overproduction could be blocked with a GABA-A receptor antagonist *in vivo*. Studies from the same group also showed that IL-4Rα is required for allergen-induced up-regulation of GABAergic system in airway epithelium, which might have a role in goblet cell metaplasia following acute house dust mite exposure [40].

Fu et al [41] showed that incubation of rhesus macaque bronchial epithelial cells with nicotine for 48 hr significantly increased mucin mRNA levels. Interestingly, the effect of nicotine was blocked both by the nicotinic antagonists and by the GABA-A receptor antagonists. This suggests that the sequential activation of nicotinic signaling followed by GABAergic signaling is necessary for nicotine to stimulate bronchial epithelial mucus production, and that nicotine-induced mucin overproduction is, in part, dependent on GABA-A receptor signaling in bronchial epithelial cells. Ly6/neurotoxin 1 (Lynx1), the founding member of a family of mammalian prototoxins, modulates nicotinic acetylcholine receptors *in vitro *by altering agonist sensitivity and desensitization kinetics [42]. Results from Lynx1 knockdown experiments suggested that Lynx1 acts as a negative modulator of nicotine-mediated activation of GABAergic signaling [43]. Interestingly, when BALB/c mice were exposed to secondhand smoke, there was an excellent correlation between increased GABA-A receptor staining and lung mucous cell metaplasia [44].

Based on the observations in the present study, there may be a therapeutic advantage to use GAD67 as a pharmacologic target for smoking-related disorders in the lung. However, while mucus overproduction is commonly associated with smoking and many COPD patients have mucus production, there is variability in the extent of mucus production among smokers and smoking-related disorders [45], any therapy focused on mucus overproduction would have to be tailored to the individual.

## Conclusions

There is a complete GABAergic system in human large and small airway epithelium. Marked up-regulation of GAD67 by cigarette smoking is associated with MUC5AC overexpression. In the context of these observations, the GABAergic system is a promising pharmacological target for inhibiting airway mucus overproduction.

## Competing interests

The authors declare that they have no competing interests.

## Authors' contributions

All authors have read and approved the final manuscript.

GW participated in study design, gene expression analysis and interpretation, statistical analyses, TaqMan RT PCR analyses and drafted the manuscript. RW participated in study design and western blot. BF participated in immuohistochemistry. JS participated in data analysis, statistical analysis. YSB participated in data analysis, statistical analysis. NH participated in gene expression analysis and interpretation, and provided helpful discussion. RGC conceived the study, oversaw collection of biological samples, participated in study design and coordination, and helped with drafting the manuscript.

## Supplementary Material

Additional file 1 **Table S1. Primer Sequences for Human GABAergic System Genes**. Table of primer sequences for human GABAergic system genes. **Figure S1. **Western analysis of GAD67 protein expression in small airway epithelium of healthy nonsmokers, healthy smokers and COPD smokers. Additional figure to support the manuscript. Click here for file
