# Supplementary material for: Smoking-mediated up-regulation of GAD67 expression in the human airway epithelium
Source: Respir Res. 2010 Oct 29;11(1):150. doi: 10.1186/1465-9921-11-150 (PMC2988726; doi:10.1186/1465-9921-11-150)
Supplement: Additional file 1 — Table S1. Primer Sequences for Human GABAergic System Genes. Table of primer sequences for human GABAergic system genes. Figure S1. Western analysis of GAD67 protein expression in small airway epithelium of healthy nonsmokers, healthy smokers and COPD smokers. Additional figure to support the manuscript. [file 1465-9921-11-150-S1.PDF]

Additional File 1, Table SI. Primer Sequences for Human GABAergic System Genes

Gene symbol	Genebank access number		Primer sequence	Amplicon size, bp	Ref
GAD65	NM_000818	F	5-GAGTGGAGTGGAGAGGGCCAACTCTGTGAC-3	488	[1]
		R	5-TTGTGGTTCCATACTCCATCATTCTGGCTTTAATC-3		[1]
GAD67	NM_000817	F	5-GACAATGTGATTTTGATAAAGTGCAATGAA-3	375	[1]
		R	5-CATCTGGTTGCATCCTTGGAGTATACCCT-3		
GABRA1	NM_000806	F	5-GAAGAGAAAGATTGGCTACTTTGTTATTCAAACAT-3	371	[2]
		R	5-GAGCGTAAGTGTTGTTTTTCTTAATAAGAGGAT-3		
GABRA2	NM_000807	F	5-TCTGCCCTAATTGAATTTGCAACTGTTAATTACTT-3	295	[2]
		R	5-CTATTCTGGACATTCTGTCAATTTTGCTAACACTG-3		
GABRA3	NM_000808	F	5-CACTTCCATCTCAAGCGAAAAATTGGCTACTTTGT-3	398	[2]
		R	5-CCCACGATGTTGAAGGTAGTGCTGGTTTTCT-3		
GABRA4	NM_000809	F	5-CAAACCGTATCAAGTGAAACCATCAAATCAAT-3	225/72[1]	[2]
		R	5-GCTTAGTGTGGTCATGGTGAGGACAGTTGTTAT-3		
GABRA5	NM_000810	F	5-GCAGACGGTGGGCACTGAGAACA-3	138	[2]
		R	5-GATAAGATCACGGTCATTATGCAGGGAAGGTA-3		
GABRA6	NM_000811	F	5-CAGTGACAATATCAAAAGCTACTGAACCTTTGGAA-3	259	[2]
		R	5-AATCCTGCAAATGCAACTGGGAAGAGAA-3		
GABRB1	X14767	F	5-TCGCACTAGGAATCACGACGGTGCTTA-3	316	[2]
		R	5-GAGCCACTCGTCTCATTCCGGATTT-3		
GABRB2	S77553	F	5-GCTGCCAGTGCCAACAATGAGAAGA-3	170	[2]
		R	5-TGGGGGTCCATCGTATACAGAGAGAAA-3		
GABRB3	M82919	F	5-TCACAACTGTGCTGACAATGACAACCATCAAC-3	474	[2]
		R	5-TAATTTTGAGCTGTGAAGACCTCCTCCGTAGA-3		
GABRG1	NM_173536	F	5-CTTTCCCATGGATGAACATTCCTGTCCACTGGAATTTT-3	320	[2]
		R	5-CAGGCACTGCATCTTTATTGATCCAAAAAGACACCC-3		
GABRG2	NM_198904,et al[1]	F	5-AGGTCTCCTATGTCACAGCGATGGATCTCT-3	267/243[1]	[2]
		R	5-GACACTCATAGCCGTACTCTTCATCTCTCTCT-3		
GABRG3	NM_033223	F	5-CGCTGATGGAGTATGCCACCCTCAACTACTATT-3	309	[2]
		R	5-GCCCTTTCCTCCAGGATCCTGATTTACATTCT-3		
GABRE	NM_004961	F	5-CACATGCTCAGATTTCCAATGGATTCTCACTCTT-3	378	[2]
		R	5-CAACGTGGTCATGGTCAGAACAGAGGTGAT-3		
GABRQ	NM_018558	F	5-GGGAAGGACGATTACTAGCAAGGAGGTGTATT-3	310	[2]
		R	5-CACAAAGAACAAGCACACGAGGATATAGATATCAA-3		
GABRD	NM_000815	F	5-GCAGTTCACCATCACCAGCTACCGCTTCAC-3	448	[2]
		R	5-GACAATGGCGTTCCTCACGTCCATCTCT-3		
GABRP	NM_014211	F	5-CACAAAGAACAAGCACACGAGGATATAGATATCAA-3	304	[2]
		R	5-GAACCTGCATTGGAGTGACGACCGTGTTA-3		
GABRR1	NM_002042	F	5-GACATCACCAAATCGCCTCT-3	277	–[2]

Additional File 1, Table SI. Primer Sequences for Human GABAergic System Genes (cont., page 2)

Gene symbol	Genebank access number	Primer sequence	Amplicon size, bp	Ref
		R 5-TGTCAGGGACCCAGATCTTC-3		
GABRR2	NM_002043	F 5-CTCGTGGAGAGCAGAAAACC-3<br>R 5-GGTGGTGTCATGAGTGAACG-3	408	–[2]
GABRR3	NM_001105580	F 5-GTTTCGGCCATGTGCTTTAT-3<br>R 5-AGGAACAGCTCTTCGGTCAA-3	354	–[2]
GABBR1	NM_001470, et al[1]	F 5-CAAGAAGATTGGCTACTATGACAGCACCAAGGATGA-3<br>R 5-CCAGGGGGAAGACAGCAGCTAAAGCCAGTGAG-3	302/150[1]	[1]
GABBR2	NM_005458	F 5-GTCCACCTCGGTCACCAGTGTGAACCAA-3<br>R 5-AGCCGACGCTGGATGTGTTCTGGAGAGT	357	[1]
GAT-1	NM_003042	F 5-GTCAAGGTGCAGAAGAAGGC-3<br>R 5-GCCAGAATGATAGCACAGCA-3	313	–[2]
GAT-2	NM_016615	F 5-CGTTCTCCTGGGACTGGATA-3<br>R 5-GAAGAGAAAGGTGGCTGTGC-3	382	–[2]
GAT-3	NM_014229	F 5-TCGCTCATTAAGTGGTGCTG-3<br>R 5-CGTCACTCTTGAGTTTGGCA-3	331	–[2]
BGT-1	NM_003044	F 5-GCCTGGGCTCTCTTCTACCT-3<br>R 5-GACTTGACCCCCTTCCAGAT-3	289	–[2]
VGAT	NM_080552	F 5-ACATCCATTATCAGCGAGGC-3<br>R 5-CACTCACCACCACGTACAGG-3	432	–[2]
GABA-T	NM_000663	F 5-ACGCATCCGATGACTTCTTT-3<br>R 5-CCTGAACTCCTCCTTGTGGA-3	206	–[2]
ALDH5A1	NM_001080	F 5-GCCGTGTTTACGGAGACATT-3<br>R 5-TCAGTACAAATTGCCTCCCC-3	307	–[2]

[1] Alternative splicing isoforms.
[2] "-": primers designed using "http://www.ncbi.nlm.nih.gov/tools/primer-blast/index.cgi"

Additional File 1, References

1. Mizuta K, Osawa Y, Mizuta F, et al. Functional expression of GABAB receptors in airway epithelium. Am J Respir Cell Mol Biol 2008;39:296-304.
2. Mizuta K, Xu D, Pan Y, et al. GABAA receptors are expressed and facilitate relaxation in airway smooth muscle. Am J Physiol Lung Cell Mol Physiol 2008;294:L1206-L1216.

Additional File 1, Figure Legends

Figure S1. Western analysis of GAD67 protein expression in small airway epithelium of healthy nonsmokers, healthy smokers and COPD smokers. **A.** Upper panel - GAD67 protein expression in nonsmokers (lanes 1-3), smokers (lanes 4-6) and COPD smokers (lanes 7-10). Middle panel - same gel probed with anti MUC5AC antibody. Lower panel - same gel probed with anti β-actin antibody; 20 μg protein loaded per well. The MUC5AC bands are broad for several reasons, including multimer formation and variable glycosylation. **B.** MUC5AC and GAD67 expression at protein level. MUC5AC or GAD67 expression (pixel intensity from digitalized image of panel A) was first normalized with β-actin, and then normalized by mean value from healthy nonsmokers group. Error bars represent standard error. **C.** Correlation of MUC5AC and GAD67 expression at protein level (Pearson's correlation). GAD67 protein expression was plotted against MUC5AC protein expression from all 10 individuals.

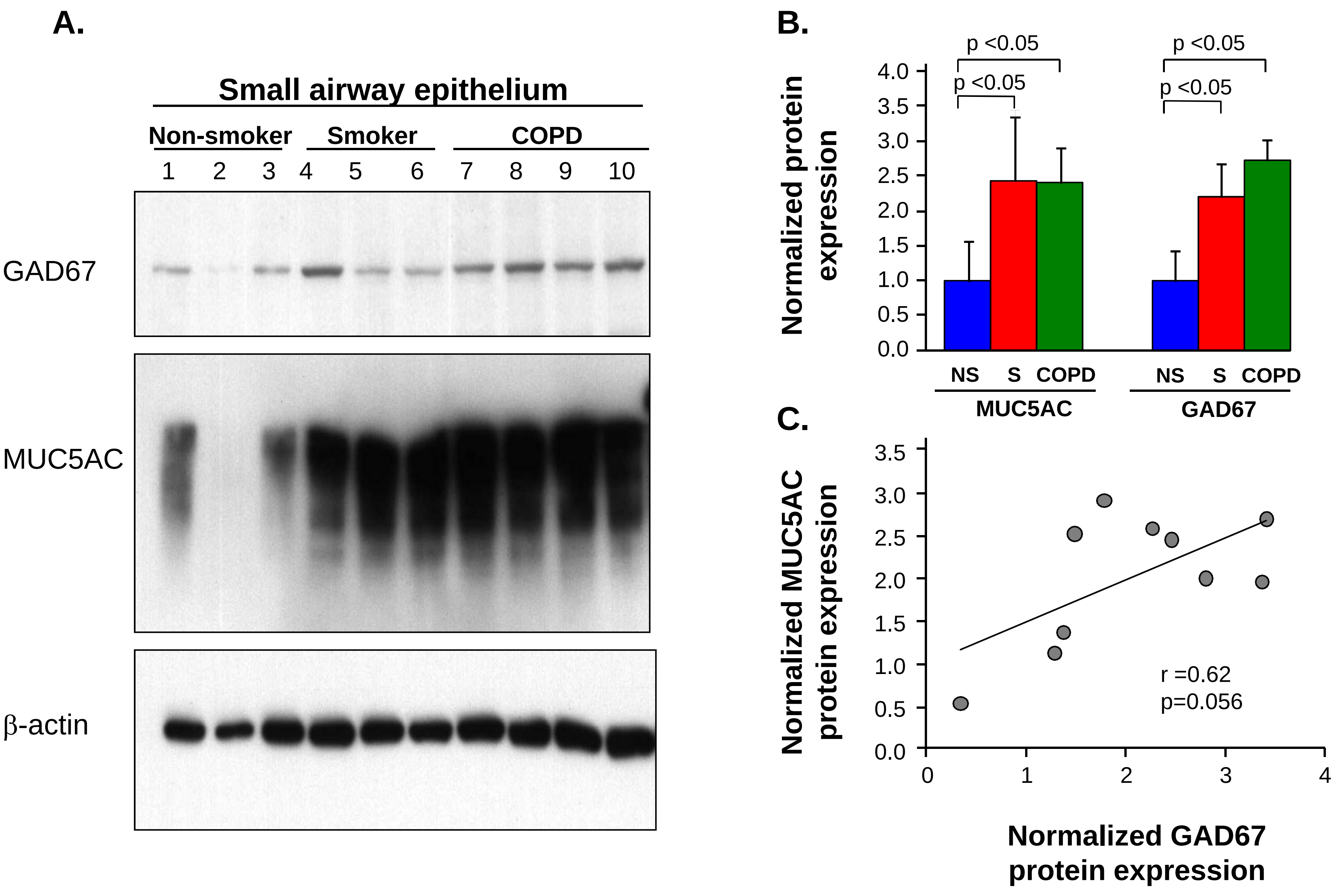

Wang et al
Figure S1
